# Steroid Injection and Nonsteroidal Anti-inflammatory Agents for Shoulder Pain: A PRISMA Systematic Review and Meta-Analysis of Randomized Controlled Trials

**DOI:** 10.1097/MD.0000000000002216

**Published:** 2015-12-18

**Authors:** Yaying Sun, Jiwu Chen, Hong Li, Jia Jiang, Shiyi Chen

**Affiliations:** Department of Sports Medicine and Arthroscopy, Huashan Hospital, Fudan University, Shanghai 200040, People's Republic of China.

## Abstract

Advantages and possible risks associated with steroid injection compared with nonsteroidal anti-inflammatory drugs (NSAIDs) for shoulder pain are not fully understood.

To compare the efficiency and safety of steroid injection versus NSAIDs for patients with shoulder pain.

PubMed, Embase, and the Cochrane Library were searched through July 2015.

Study eligibility criteria, participants, and interventions: randomized controlled trials (RCTs) that assessed steroid injection versus NSAIDs for patients with shoulder pain.

Study appraisal and synthesis methods: predefined primary efficacy outcome was functional improvement; and secondary efficacy outcomes included pain relief and complications. Relative risks (RRs) and standardized mean differences (SMDs) with 95% confidence intervals (CIs) were calculated using a random-effects model accounting for clinical heterogeneity.

Eight RCTs involving 465 participants were included in the meta-analysis. Five trials compared steroid injection with oral NSAIDs, and 3 compared steroids injection with NSAIDs injection. Compared with steroid injection, oral NSAIDs were less effective in 4 or 6 weeks for functional improvement (SMD 0.61; 95% CI, 0.08–1.14; *P* = 0.01), while there was no significant difference in pain relief (SMD 0.45; 95% CI, −0.50–1.40; *P* < 0.00001) or complication rate (RR 1.10; 95% CI, 0.26–4.58; *P* = 0.29). Meta-analysis was not performed for NSAIDs injection due to considerable heterogeneity. Conflicting results were observed in favor of either steroid or NSAIDs injection.

Not all diseases that can lead to shoulder pain were included, detailed intervention protocols were inconsistent across studies, and some estimated data were input into comparison while some data were lost, which could exert an influence on pooled results.

Steroid injection, compared with oral NSAIDs, provides slightly more improvement in shoulder function without superiority in pain relief or risk of complications at 4 to 6 weeks.

Treatment decision should be made based on diseases. NSAIDs injection might be a treatment method for shoulder pain.

## Abstract 2

Supplemental Digital Content is available in the text

## INTRODUCTION

Shoulder pain is an important medical problem in the world, with a prevalence between 7% and 26% of the population at any one time.^1^ Pain and subsequent dysfunction in the shoulder leads to disability, thus decreasing the quality of life. Manyconditions, such as adhesive capsulitis, tendinitis, and shoulderimpingement syndrome (SIS), can result in shoulder pain.^2–5^ Inflammation, which causes glenohumeral pain and tissue degeneration, is usually the factor underlying this complaint.

Based on these recognitions, many nonoperative modalities, including steroid injection, nonsteroidal anti-inflammatory drugs (NSAIDs), and shockwave, are introduced into practice and have accumulated considerable experience.^6,7^ Steroid injection has long been used for shoulder pain, relying mainly on its strong anti-inflammation effect. However, side effects, such as pain, vasovagal reaction, serum glucose level changes, and facial flush reaction, might prevent patients from this treatment.^8–10^ Compared with steroid injection, NSAIDs might provide similar pharmacological effect with less adverse effect, thus encouraging the administration to patients.^11,12^

Previously, 2 meta-analyses have summarized the evidence on this topic. The conclusion, however, is controversial. A meta-analysis involving only 3 trials conducted by Arroll and Goodyear-Smith^13^ found no difference between steroid injection and NSAIDs for shoulder pain, while Zhang et al^14^ observed a significant superiority of steroid injection to NSAIDs, after analyzing 6 randomized controlled trials (RCTs). Therefore, based on newly published articles,^15,16^ an update review is needed.

## METHODS

The systematic review was written in adherence to the PRISMA (Preferred Reporting Items for Systematic Reviews and Meta-analyses) checklist.^17^ Ethical approval was not necessary according to local legislation because of the type of study (meta-analysis).

### Search Strategy

Electronic search was performed independently by the first 2 authors through July 2015 on Pubmed, Embase, Cochrane Central Registers of Controlled Trials, and Cochrane Database of Systematic reviews. Reference lists of previous systematic reviews with regard to physiotherapy in adhesive capsulitis and the included studies were also reviewed. Detailed searching strategy for Pubmed is in Appendix.

### Inclusion Criteria

RCTs comparing the effect of steroid injection with NSAIDs for patients with shoulder pain were included. No language filter was performed.

### Type of Outcome Measures

The primary outcome of interest was functional improvement, for example, Shoulder Pain and Disability Index or The American Shoulder and Elbow Surgeons score. Secondary outcomes were pain relief and complication rate. Range of motion was not selected as one of the outcome of interest because different diseases had different characteristic limitation of range of motion. Comparisons were performed at 4 to 6 weeks after intervention as this period was also the commonly applied course of oral NSAIDs.

### Study Selection

The first 2 authors independently reviewed all titles included after primary literature research. Inconsistencies were resolved by discussion and consensus.

### Data Collection and Management

The first 2 reviewers independently extracted the outcomes of interest and complication rate from the included studies. Besides, the author and published year, disease, number of patients included, interventions details, and summary of findings were extracted. Any disagreement would be resolved by discussion and consensus.

### Data Analysis

Review Manager, Version 5.3 (The Nordic Cochrane Centre, The Cochrane Collaboration; Copenhagen, Denmark) was used for all analyses. A 2-tailed *P* value < 0.05 was regarded as statistically significant. A random-effects model was used for comparisons because disease categories, disease duration, detailed intervention protocols, and other confounding factors were inconsistent among studies. However, heterogeneity was also assessed by Q statistic and I^2^ statistic. The latter describes the percentage of total variation across studies due to heterogeneity rather than chance. Significant heterogeneity was defined as an I^2^ statistic larger than 40%. Standardized mean differences (SMDs) and 95% confidence intervals (CIs) for pain relief and functional improvement were calculated since inconsistent measure methods were used in different studies. An effect size of 0.2 was considered small beneficial effect, 0.5 was considered a medium effect and more than 0.8 was considered as a large effect.^18^ Relative risks with 95% CI was used to calculate the difference of complications. When standard deviation (SD) was known for baseline and endpoint instead of change, a correlation of 0.5 was used to estimate the dispersion.^19^ When SD was not reported and could not be calculated from available data, we asked authors to provide the data. In the absence of data from authors, mean SD calculated from available studies was put to use. Publication bias was not detected due to limited number of studies included.^20^ Subgroups would be introduced into analysis according to different diseases. Specifically, tendinitis would be regarded as SIS.^21^ Whenever heterogeneity was significant, sensitivity analysis was performed. One study would be omitted in each turn to figure out the origin of heterogeneity.

### Assessments of Quality of Evidence

The first 2 reviewers independently used the Cochrane risk of bias tool to evaluate the risk of bias of each included trial.^22^ A value of low, unclear, or high risk of bias was assigned to the following items: random sequence generation, allocation concealment, blinding of participants and personnel, blinding of outcome assessment, incomplete outcome data, selective reporting, and other bias. Any inconformity will be resolved by discussion. The overall quality of evidence for each of the outcomes was rated by applying the Grading of Recommendations Assessment, Development, and Evaluation approach.^23^ Grading of Recommendations Assessment, Development, and Evaluation Working Group grades of evidence were as follows: high quality, moderate quality, low quality, and very low quality. Publication bias was not able to assess and therefore was rate as none. Specifically, evidence would be downgraded if heterogeneity was larger than 40%.^24^

## RESULTS

After removing duplicates, 275 studies were identified by primary search. After reading titles and abstracts, 266 were excluded and 9 were left. Full text of the 9 articles were reviewed for further evaluation. Two were excluded due to the employment of additional treatment modalities in NSAIDs group.^25,26^ One study was identified to be eligible by reading reviews.^15^ Finally, 8 articles were included (Table 1).

**TABLE 1:**
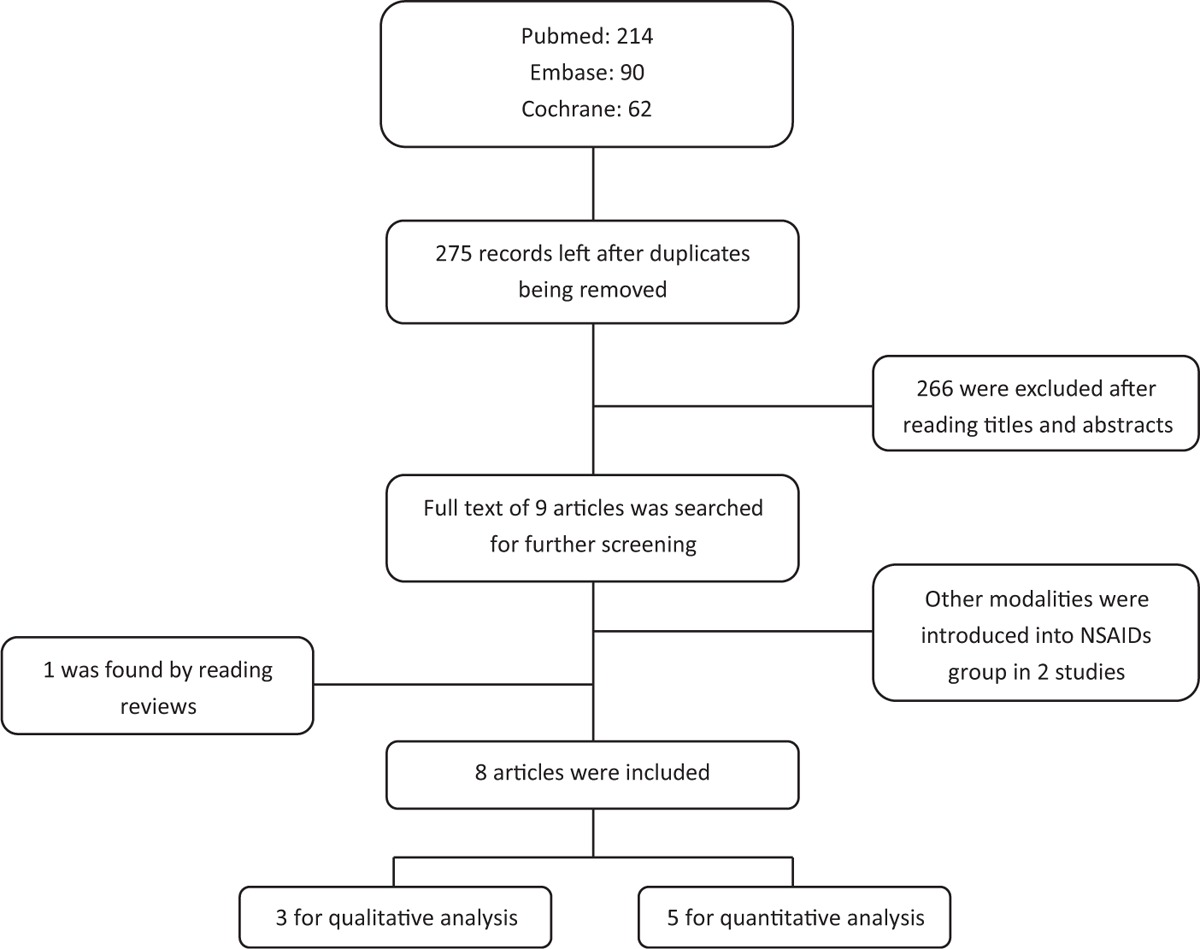
The table of new edition is listed below

Five studies compared steroid injection with oral NSAIDs and were analyzed in a quantitative manner.^11,15,27–29^ Three compared steroid injection with NSAIDs injection and were analyzed in a qualitative manner.^16,30,31^ Basic information of included studies are listed in Table 2. A total of 465 patients were included in the current analysis, of which 273 received steroid injection. All steroid injections were performed only once. Anesthetics was not reported to be combined with steroid injection in 2 studies^27,29^ and not combined with NSAIDs injection in 1 study.^16^ Specific drug utilization is listed in Table 2. Among the included studies, 2 were adhesive capsulitis,^15,29^ 1 was nonspecific painful shoulder,^28^ 2 were tendinitis,^11,27^ and 3 were SIS.^16,30,31^

**TABLE 2:**
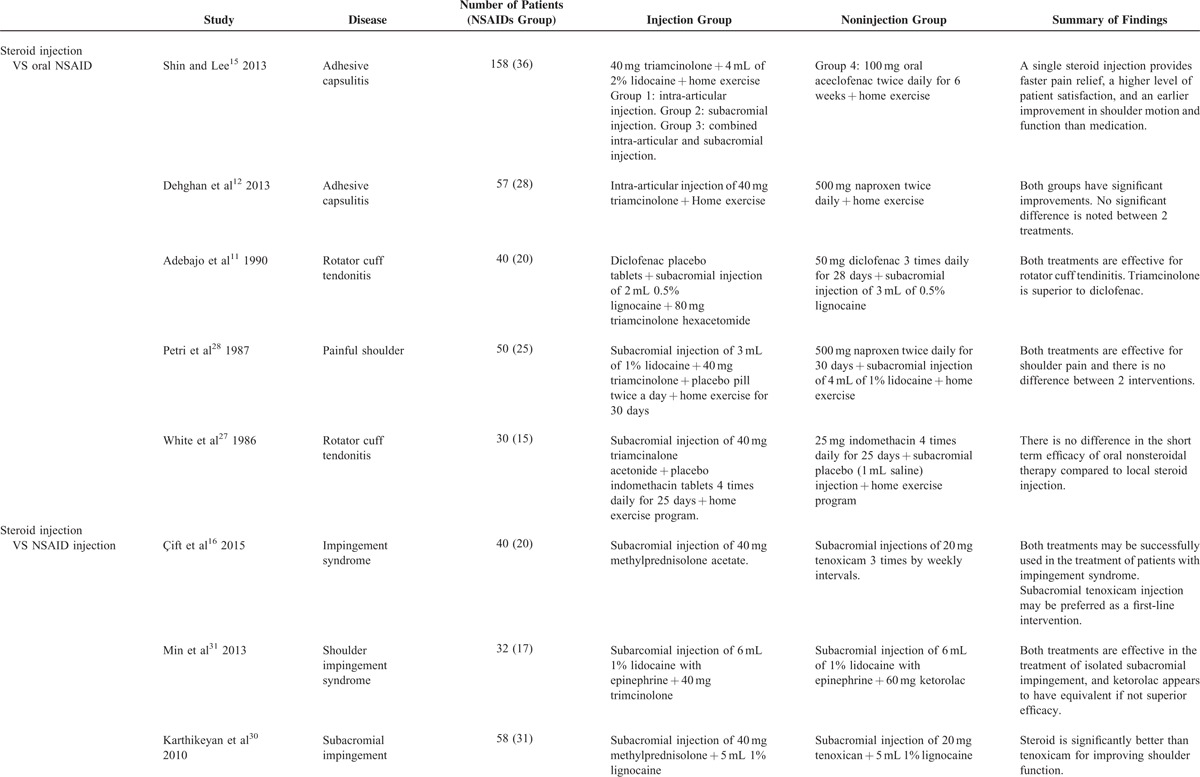
Basic Information of Included Studies

**FIGURE 1:**
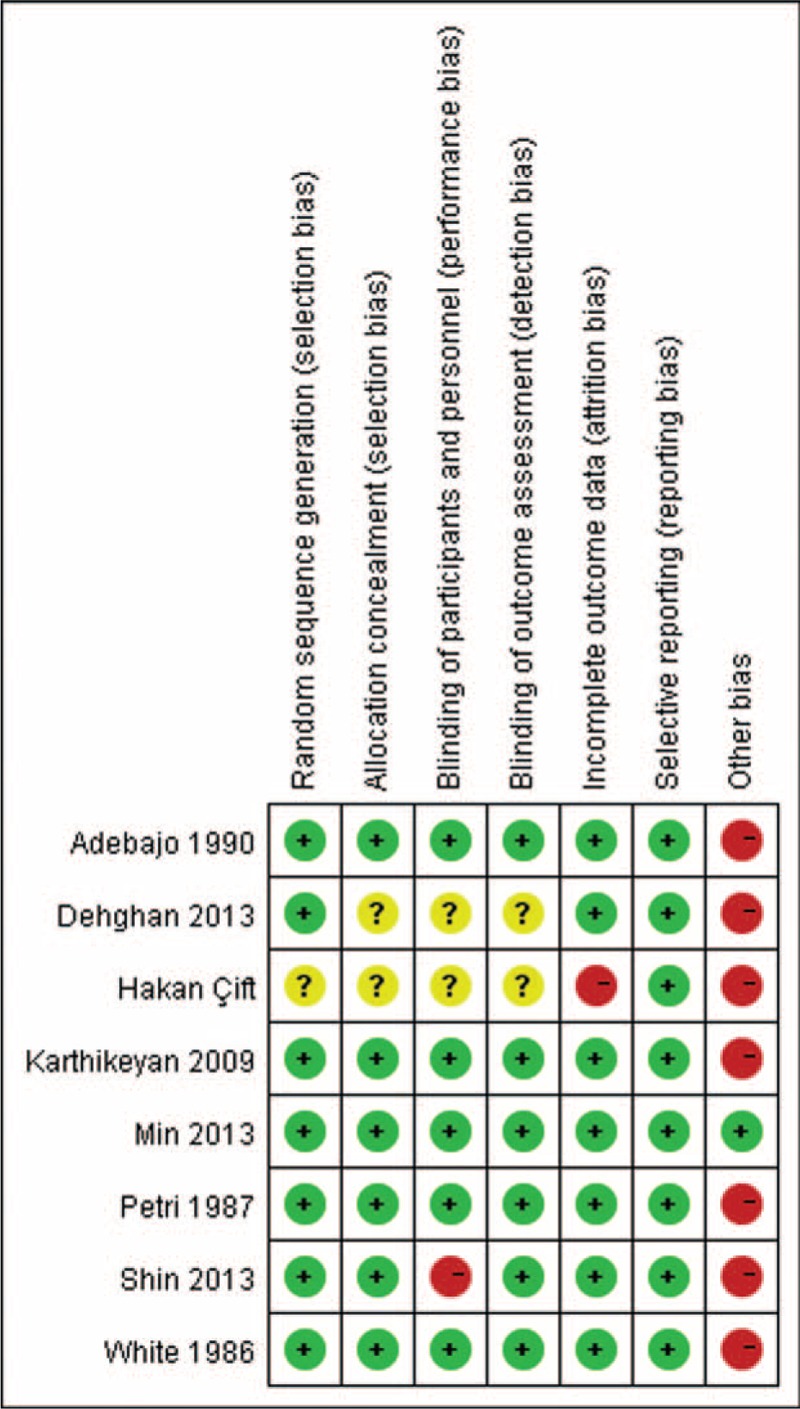
Risk of bias summary.

The risk of bias of each study is shown in Figure 1. No study employed intention-to-treat method and only 3 studies had sample size calculation prior to interventions.^15,30,31^ No patient was lost follow-up in 2 of the studies.^11,28^ Two studies did not described the allocation concealment and blinding of participants.^16,29^ Patients were not blinded to treatments in 1 study.^15^ In 1 study, the data at 6 weeks were not available.^16^

### Steroid Injection Vs Oral NSAIDs

A total of 231 patients received steroid injection while 124 were administered with oral NSAIDs. Injection dose was 40 mg in 4 studies and 80 mg in 1.^11^ The dose of oral NSAIDs ranged from 100 to 1000 mg/daily, frequency ranged from twice a day to 4 times a day, and duration ranged from 25 days to 6 weeks.^15,27^ Duration was not reported in 1 study^29^ (Table 2).

### Functional Improvement

**FIGURE 2:**
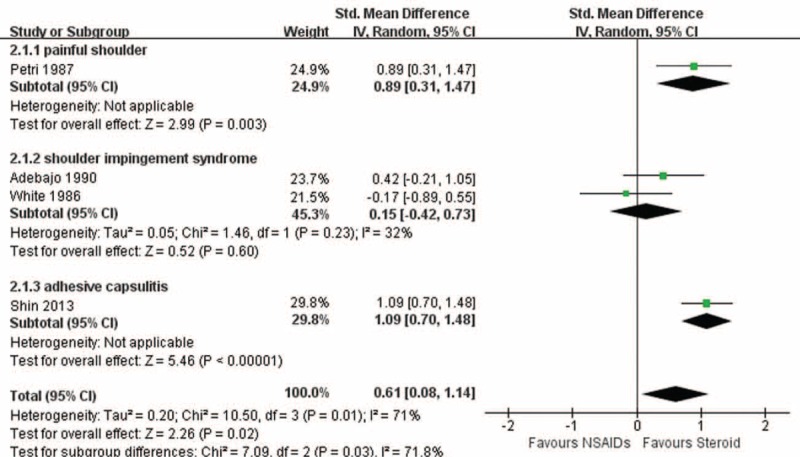
Functional improvement.

Four studies with 3 diseases reported data in terms of functional improvement.^11,15,27,28^ The pooled result showed superiority in favor of steroid injection (SMD 0.61; 95% CI, 0.08–1.14) with significant heterogeneity (I^2^ = 71%, *P* = 0.01) (Fig. 2). The level of evidence was low. Subgroup analysis found that NSAIDs, compared with steroid injection, had similar results for SIS but was inferior to steroid injection for nonspecific shoulder pain and adhesive capsulitis. The heterogeneity was not significant (I^2^ = 37%, *P* = 0.21) with a pooled result in favor of steroid injection (SMD 0.86; 95% CI, 0.48–1.24) after omitting 1 study from comparison.^28^ Subsequently, the corresponding level of evidence changed from low to moderate.

### Pain Relief

**FIGURE 3:**
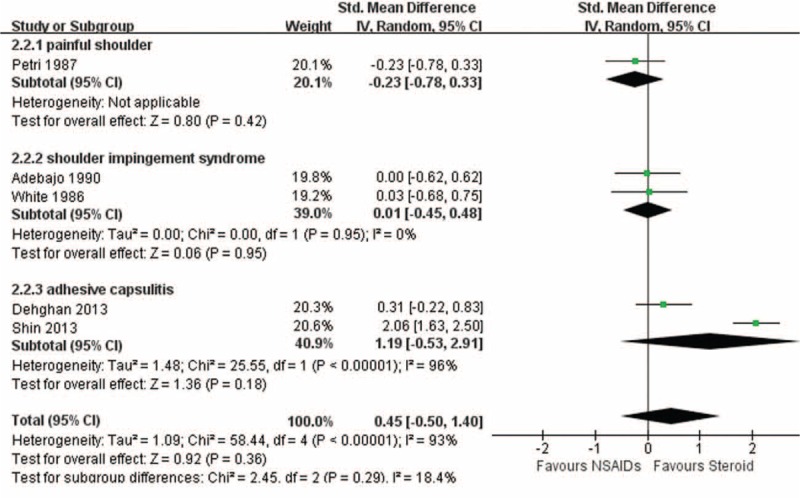
Pain relief.

Five studies with 3 diseases had results in view of pain relief.^11,15,27–29^ The pooled result showed no superiority in favor of either treatment (SMD 0.45; 95% CI, −0.50–1.40) with significant heterogeneity (I^2^ = 93%, *P* < 0.00001) (Fig. 3). The level of evidence was low.

### Complication Rate

**FIGURE 4:**
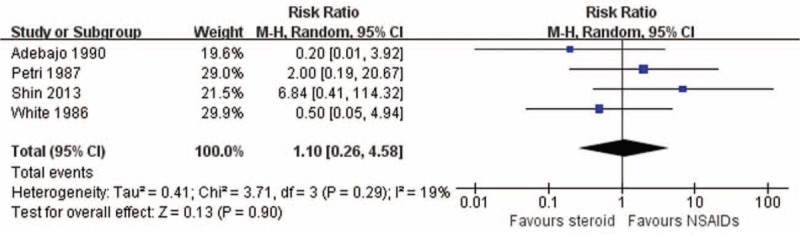
Complication rate.

Four studies reported complications.^11,15,27,28^ The most commonly reported complications were skin color change and facial flushing reaction due to injection. Two gastrointestinal reaction, 1 headache, and 2 dyspepsia were identified.^11,27,28^ The pooled result showed no superiority in favor of either treatment (RR 1.10; 95% CI, 0.26–4.58) with no significant heterogeneity (I^2^ = 19%, *P* = 0.29) (Fig. 4).

### Steroid Injection Vs NSAIDs Injection

Three studies compared steroid injection with NSAIDs injection.^16,30,31^ Sixty-eight patients received NSAIDs injection while 62 received steroid injection. Injection frequency ranged from 1 to 3, and dosages ranged from 20 to 60 mg (Table 2). The summary of results is listed in Table 3. Data at 6 weeks after interventions were not available, and authors could not be connected in 1 article.^16^ One study found that 20 mg NSAIDs injection was inferior to 40 mg steroid injection in improving function for as long as 6 weeks, with high level of evidence.^30^ One study found that 60 mg NSAIDs injection was equally effective to 40 mg steroid injection in improving function and relieving pain for 4 weeks, with high level of evidence.^31^ One study did not report data at 6 weeks. In this study, 20 mg NSAIDs injection was performed 3 times at weekly interval and was found to be significantly more beneficial than a single 40 mg steroid injection in improving function and relieving pain for 1 year.^16^ The corresponding level of evidence was moderate. Only 1 complication, a temporary fainting episode, was found in steroid injection group.^31^

**TABLE 3:**
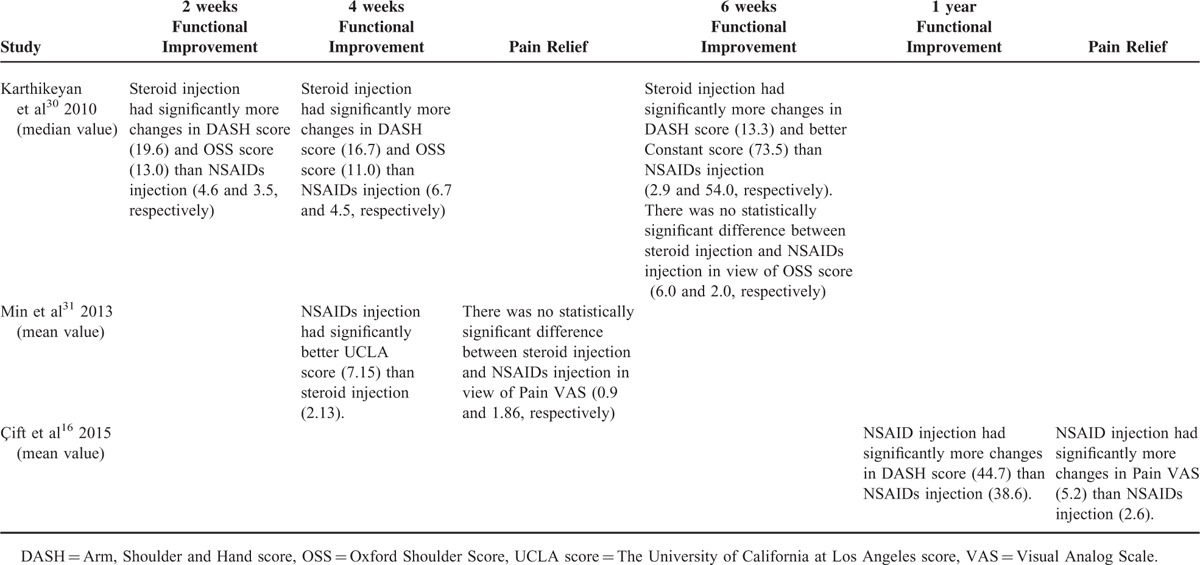
Steroid Injection Versus NSAIDs Injection

## DISCUSSION

This is a further meta-analysis about the effect of steroid injection versus NSAIDs for shoulder pain. The present study of 8 RCTs showed that compared with oral NSAIDs, steroid injection could provide significantly more functional improvement for painful shoulder, albeit with similar effect on pain relief. Complication was observed without superiority in favor of either treatment, indicating equal safety for both interventions. When steroid injection was compared to injectable NSAIDs, the conflicting results indicated the remarkable differences in study design and intervention protocols. Injectable NSAIDs might be a reasonable choice for shoulder pain.

Two former meta-analyses were conducted on this topic. Arroll and Goodyear-Smith^13^ compared steroid injection versus oral NSAIDs in terms of remission rate, a predefined method to reflect the number of patients who had evident response to treatment, and did not find any superiority in favor of either intervention. In addition to remission rate, another meta-analysis chose pain relief and active abduction as secondary outcomes and found that steroid injection was superior to NSAIDs with significantly higher remission rate with similar effect on pain relief and active abduction.^14^ However, different administration methods, that is, oral and injectable NSAIDs, were applied in the included studies and were not distinguished from each other in the pooled results. In our study, instead of remission rate, we employed the mean and SD of functional improvement as the primary outcome, since remission rate, as a dichotomous value, could not show the improvement of glenohumeral function, which was a continuous progress, and was defined inconsistently across studies.^11,27,28,30^ Besides, different diseases had different characteristic loss of motion. Adhesive capsulitis causes loss of passive external rotation, while SIS leads to abduction defect.^32^ On ground of this heterogeneity, active abduction was not an outcome of interest in our study. Instead, we employed the complication rate as another secondary outcome, in an attempt to figure out the safety of both treatments. According to the pooled results, both treatments had similar complication rate, and complications were mainly temporary and not serious. Besides, injectable and oral NSAIDs were compared to steroid injection separately, providing more detailed evidence for clinical practice.

The heterogeneity was remarkably significant in functional improvement. This was mainly caused by inconsistent intervention protocols and different diseases, the latter of which was analyzed by subgroup analysis. The heterogeneity was caused by the study conducted by White et al.^27^ In this study, 25% of patients (5 in each group) were lost follow-up, which could exert an influence on the final outcomes, according to intention-to-treat method.

Characterized by gradual loss of passive external rotation and shoulder pain, adhesive capsulitis is one of the most common musculoskeletal problems seen in orthopedics, and has a prevalence greater than 2% in the general population.^33^ SIS, or SAIS, is the most frequently reported diagnosis of shoulder pain, representing a spectrum of diseases ranging from tendinitis to partial or full-thickness rotator cuff tears that affect daily overhead activities.^21^ In the comparison of steroid injection versus oral NSAIDs, 2 diseases, that is, adhesive capsulitis and SIS, were both introduced into comparison and each had 2 studies included, increasing the strength of this comparison. Specifically, for SIS, oral NSAIDs were proven to be an alternative treatment to steroid injection, with high level of evidence.

Different dosages were used for shoulder pain. For SIS a dosage ranging from 100 to 150 mg/day was proven to be as effective as steroid injection,^11,27^ while for adhesive capsulitis the dosage that could lead to similar effect to steroid injection was 1000 mg/day,^29^ indicating the differences of inflammation in scale and degree between 2 diseases. An accurate diagnosis is of vital importance to guide clinical practice.

Concern must be taken in relate to oral NSAIDs, which carries significant dose-related risks of cardiovascular, renal, hematological, and other systemic adverse events especially for the elderly, who are more likely to suffer shoulder pain.^34^ In current included studies, oral NSAID-related adverse effects were identified. In order to reduce these risks, high risk patients should be notified, and protective drugs concomitant with less damaging NSAIDs should be prescribed at the lowest effective therapeutic dose for the shortest possible duration.^35,36^ However, different dosages, frequencies, and durations indicated that there was no consensus in the administration of oral NSAIDs. Therefore, as an effective and efficient means to reduce systemic NSAIDs exposure, topical NSAIDs, especially injectable NSAIDs, was introduced into clinical use.

In the current study, all NSAIDs injections were performed into subacromial area for SIS. According to the available evidence, different NSAIDs injection doses and frequencies were used and contradictory results were observed. Compared with 40 mg steroid injection, a single injection of 20 mg NSAIDs had less treating effect, while the same dose with a frequency of 3 times weekly was more effective.^16^ When a larger dose of injectable NSAIDs was used (60 mg), both injection methods had similar effect for SIS.^37^ Therefore, NSAIDs injection might be an option for shoulder pain, especially for SIS, though detailed injection protocols are still unclear.

There are several limitations in the current meta-analysis. First, not all diseases that can lead to shoulder pain were included, thus decreasing the reliability. Besides, although oral and injectable NSAIDs were compared to steroid injection separately, detailed intervention protocols were inconsistent across studies, undermining the current outcomes. Finally, some estimated data were input into comparison and some data were lost, which could exert an influence on pooled results.

Based on current evidence for shoulder pain, steroid injection, compared with oral NSAIDs, provides slightly more improvement in shoulder function without superiority in pain relief or risk of complications at 4 to 6 weeks. Treatment decision should be made based on diseases. NSAIDs injection might be a treatment method for shoulder pain.

## Supplementary Material

Supplemental Digital Content
