# Supplementary material for: Steroid Injection and Nonsteroidal Anti-inflammatory Agents for Shoulder Pain: A PRISMA Systematic Review and Meta-Analysis of Randomized Controlled Trials
Source: Medicine (Baltimore). 2015 Dec 18;94(50):e2216. doi: 10.1097/MD.0000000000002216 (PMC5058904; doi:10.1097/MD.0000000000002216)
Supplement: Supplemental Digital Content [file medi-94-e2216-s001.docx]

(anti-inflammatory OR non-steroidal OR nonsteroid OR acetylsalicyl* OR carbasalaatcalcium OR diflunisal OR aceclofenac OR alclofenac OR diclofenac OR indometacin OR sulindac OR meloxicam OR piroxicam OR dexibuprofen OR dexketoprofen OR fenoprofen OR flurbiprofen OR ibuprofen OR ketoprofen OR naproxen OR tiapro* OR metamizol OR tenoxicam OR phenylbutazone OR phenazone OR propyphenazone OR celecoxib OR etoricoxib OR nabumetone OR parecoxib) and (corticosteroid OR steroid OR glucocorticosteroid) and (shoulder pain OR impingement OR rotator cuff OR bursitis OR adhesive capsulitis or frozen shoulder) and (randomized controlled trial[pt] OR randomized controlled trials[mh] OR random allocation[mh] OR random allocat*[tw] OR randomly allocat*[tw] OR double-blind method[mh] OR single-blind method[mh] OR blind* [tw] OR clinical trial[pt] OR clinical trials[mh] or random*)
